# 

**Published:** 1893-08-26

**Authors:** 


CONTENTS.

**Temperature, Food, and Dress** - - - - - - - - 337

**Around the Hospitals** - - - - - - - - - 338

**The Attitude of Great Writers Towards Disease.**—Pepys on the Great Plague - - - - - - - - - - - 339

**Contemporary Theory and Research** - - - - - 340

**Famous Poisoners in Fiction.** III.—Catherine - - - 341

**Wasps' Nurseries** - - - - - - - - - - 341

**Gleanings in Science and Medicine** - - - - - 342

**Annotations.**—The Home or the Workhouse ?—Where are the British ?—Moral and Physical Analogy - - - - - - 343

**Notes and News** - - - - - - - - - - - 344

**The London and Counties Medical Protection Society (Limited)** - - - - - - - - - - - - 348

**The Hospital Clinic**—

Royal Infirmary, Edinburgh — Treatment of Cleft Palate - 345

City Road Chest Hospital.—The Treatment of Bronchitis - 346

Chronic Intraventricular Effusion of Chronic Hydrocephalus - - - - - - - - - - - - - 347

**The Institutional Workshop**—

Hospital Administration.—II.—Special Hospitals - - - 349

Hospitals in India - - - - - - - - - - 350

Practical Departments.—Gas Stoves, &c.—Emergency Case 351

Construction Notes - - - - - - - - - - 351

**Scraps and Gleanings** - - - - - - - - - - 352

**The Book World of Medicine and Science** - - - - vii

**Medical Vacancies and Appointments** - - - - - xi

EXTRA SUPPLEMENT.—THE NURSING MIRROR.

**News from the Nursing World.**—Nursing Sisters—Clothing for Infectious Hospitals—Workhouse Infirmary Nursing — Voluntary and Involuntary Alms — Clapham Maternity Hospital—Hints—Workhouse Nursing at Leeds—Low Fees and Inefficiency—Nursing at Lynn—The First Certificate—Nurses as Missionaries—The Indian Nursing Service—Short Items - - - - - - - - - ccxi
**Notes of a Lecture on Cholera.** Delivered by Mrs. Scharlieb, M.D. By One of the Audience - - - - - - - ccxiii
**A Useful Association** - - - - - - - - ccxiii
**On "Giving In."** By a Nurse - - - - - - - ccxiv
**Nurses' Homes.** II.—Present - - - - - - - ccxiv
**Everybody's Opinion.**—The Parish Nurse—Cornish Holiday Resorts—A Nurse's Home in Paris—Cottage Nurses - - ccxv
**The World of Nurses.** As Seen by an Unprofessional Correspondent. II.—A Calling or a Profession ? - - - ccxvi
**For Reading to the Sick.**—The Faint Heart - - - - ccxvii
**Minor Appointments—Wants and Workers** - - - ccxvii
**Notes and Queries** - - - - - - - - - ccxvii
**The Muses' Looking Glass.**—An International Kiss - - ccxviii
**Nurses and Cholera** - - - - - - - - - ccxviii
**The Book World for Women and Nurses** - - - ccxix

